# 6-(3-Chloro­phen­yl)imidazo[2,1-*b*][1,3,4]thia­diazole

**DOI:** 10.1107/S1600536812049793

**Published:** 2012-12-12

**Authors:** A.S. Praveen, Jerry P. Jasinski, Shannon T. Krauss, H. S. Yathirajan, B. Narayana

**Affiliations:** a Department of Studies in Chemistry, University of Mysore, Manasagangotri, Mysore 570 006, India; b Department of Chemistry, Keene State College, 229 Main Street, Keene, NH 03435-2001, USA; c Department of Studies in Chemistry, Mangalore University, Mangalagangotri 574 199, India

## Abstract

In the title compound, C_10_H_8_ClN_3_S, the dihedral angle between the mean planes of the benzene and imidazo[2,1-*b*][1,3,4]thia­diazole rings is 6.0 (9)°. In the crystal, mol­ecules are assembled by the formation of centrosymmetric dimers by π-stacking of the thia­diazole and benzene rings of neighboring mol­ecules [centroid–centroid distance = 3.6938 (11) Å] along [010].

## Related literature

For related imidazothia­diazole derivatives and their pharmacological potential, see: Palagiano *et al.* (1995 ▶). For related structures, see: Banu *et al.* (2011*a* ▶,*b* ▶); Fun *et al.* (2011*a* ▶,*b* ▶). For standard bond lengths, see Allen *et al.* (1987 ▶). 
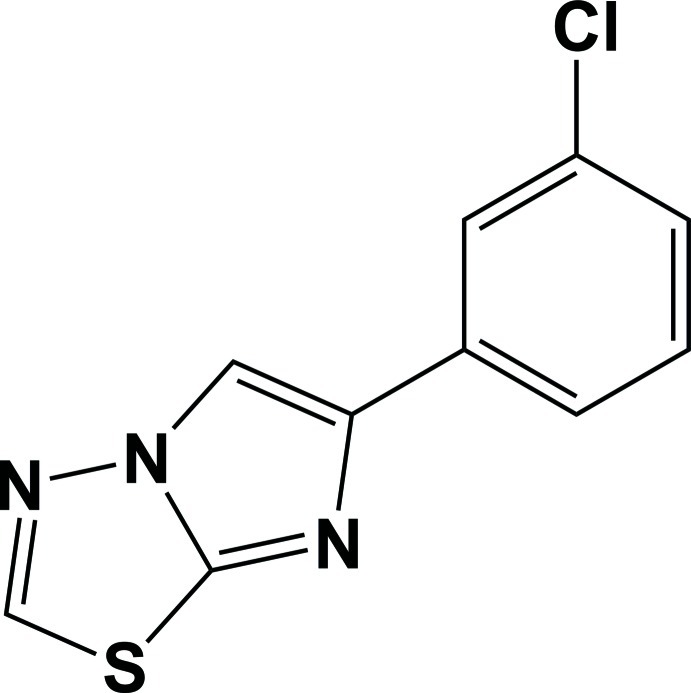


## Experimental

### 

#### Crystal data

C_10_H_6_ClN_3_S
*M* *_r_* = 235.70
Monoclinic, 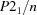

*a* = 5.43804 (19) Å
*b* = 12.4222 (4) Å
*c* = 14.1684 (4) Å
β = 100.269 (3)°
*V* = 941.77 (5) Å^3^
*Z* = 4
Cu *K*α radiation
μ = 5.37 mm^−1^
*T* = 173 K
0.26 × 0.12 × 0.06 mm

#### Data collection

Agilent Xcalibur (Eos, Gemini) diffractometer
Absorption correction: multi-scan (*CrysAlis PRO* and *CrysAlis RED*; Agilent, 2012 ▶) *T*_min_ = 0.691, *T*_max_ = 1.000
5495 measured reflections
1846 independent reflections
1650 reflections with *I* > 2σ(*I*))
*R*_int_ = 0.046

#### Refinement

*R*[*F* ^2^ > 2σ(*F* ^2^)] = 0.036
*wR*(*F* ^2^) = 0.100
*S* = 1.06
1846 reflections
137 parameters
H-atom parameters constrained
Δρ_max_ = 0.36 e Å^−3^
Δρ_min_ = −0.23 e Å^−3^

### 

Data collection: *CrysAlis PRO* (Agilent, 2012 ▶); cell refinement: *CrysAlis PRO*; data reduction: *CrysAlis RED* (Agilent, 2012 ▶); program(s) used to solve structure: *SHELXS97* (Sheldrick, 2008 ▶); program(s) used to refine structure: *SHELXL97* (Sheldrick, 2008 ▶); molecular graphics: *SHELXTL* (Sheldrick, 2008 ▶) and *Mercury* (Macrae *et al.*, 2006 ▶); software used to prepare material for publication: *SHELXTL*.

## Supplementary Material

Crystal structure: contains datablock(s) global, I. DOI: 10.1107/S1600536812049793/im2414sup1.cif

Structure factors: contains datablock(s) I. DOI: 10.1107/S1600536812049793/im2414Isup2.hkl

Supplementary material file. DOI: 10.1107/S1600536812049793/im2414Isup3.cml

Additional supplementary materials:  crystallographic information; 3D view; checkCIF report

## Figures and Tables

**Fig. 1.**
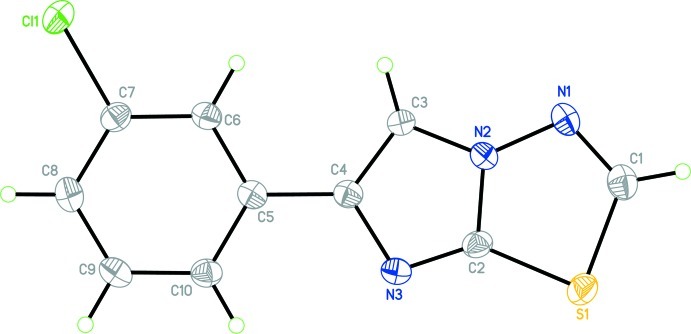
Molecular structure of the title compound showing the atom labeling scheme and 50% probability displacement ellipsoids.

**Fig. 2.**
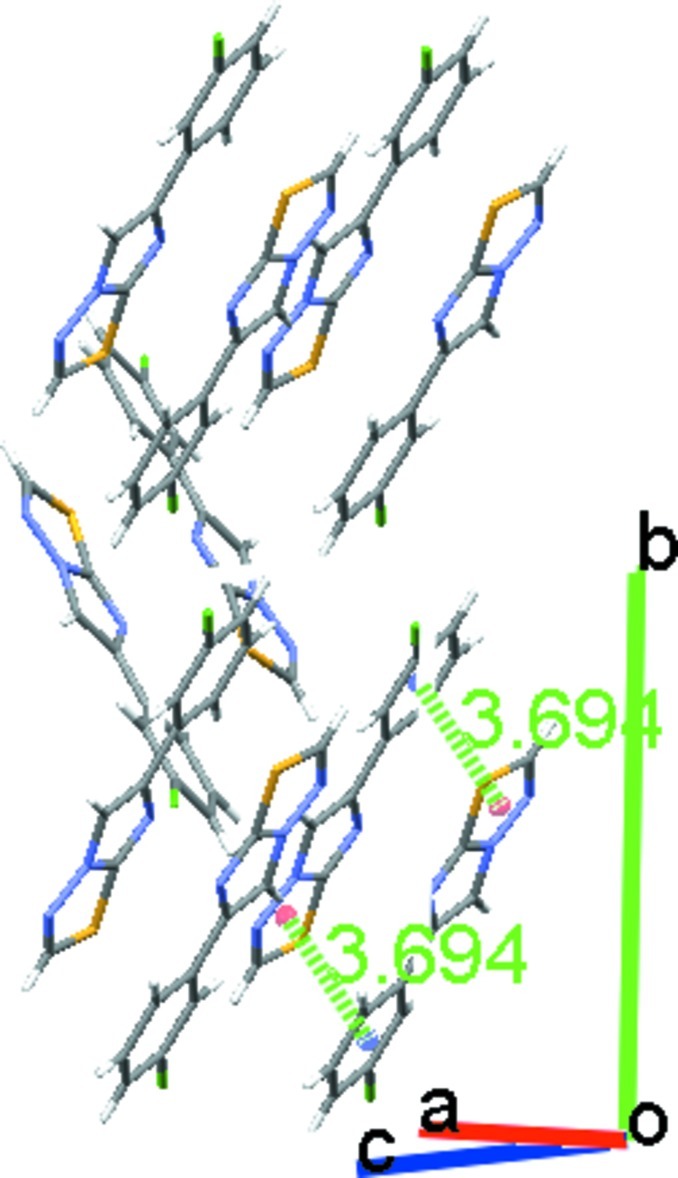
Packing diagram of the title compound viewed along the a axis. Dashed lines indicate π-stacking of thiadiazole and benzene rings of neighboring molecules (centroid–centroid distance = 3.6938 (11)Å) into centrosymmetric dimers along [010]. Hydrogen atoms have been removed for clarity.

## supplementary crystallographic information

### Comment

Many imidazothiadiazole derivatives have been reported to possess diverse medicinal properties such as anthelmintic, antimicrobial, anti-inflammatory, antipyretic, analgesic properties and many other activities of therapeutic significance (Palagiano *et al.*, 1995). The crystal structures of some imidazothiadiazole molecules, viz., 6-(4-bromophenyl)-2-(4-fluorobenzyl)imidazo[2,1-b][1,3,4] thiadiazole, 3-{[6-(4-Chlorophenyl)imidazo[2,1-b][1,3,4]thiadiazol-2-yl] methyl}-1,2-benzoxazole (Banu *et al.*, 2011*a*,*b*), 2-isobutyl-6-(4-methoxyphenyl)imidazo[2,1-b][1,3,4]thiadiazole and 6-(4-chlorophenyl)-2-isobutylimidazo[2,1-b][1,3,4]thiadiazole (Fun *et al.*, 2011*a*,*b*) have been reported.

In the title compound, C_10_H_8_N_3_SCl, the dihedral angle between the mean planes of the benzene and imidazo[2,1b][1,3,4]thiadiazole rings is 6.0 (9)° (Fig. 1). Bond lengths are in normal ranges (Allen *et al.*, 1987). In the crystal, molecules are assembled by the formation of centrosymmetric dimers by π-stacking of thiadiazole and benzene rings of neighboring molecules (centroid–centroid distance = 3.6938 (11) Å) along [010] (Fig. 2). Additional weak π-stacking interactions involving nearby benzene rings (centroid-centroid distance = 3.7739 (11) Å) are also observed.

### Experimental

A solution of 1,3,4-thiadiazol-2-amine (500 mg, 4.9 mmol) and 2-bromo-1-(3-chlorophenyl)ethanone (1.1 g, 4.9 mmol) in DMF (10 mL) was placed in a microwave pyrex tube which was introduced into a Biotage Initiator-microwave reactor fitted with a rotational system. Microwave irradiation was performed for 10 minutes at 373 K, then the mixture was cooled to ambient temperature. The reaction mass was poured into ice, the precipitated solid was filtered and dried. The single crystal was grown from a solution in ethyl acetate by slow evaporation of the solvent (yield: 97%; (m.p.: 437–440 K).

### Refinement

All H atoms were placed in their calculated positions and then refined using the riding model with C—H lengths of 0.93Å. Isotropic displacement parameters for hydrogen atoms were set to 1.18-1.21 (CH) times *U*_eq_ of the parent atom.

### Crystal data

**Table:**

C_10_H_6_ClN_3_S	*F*(000) = 480
*M**_r_* = 235.70	*D*_x_ = 1.676 Mg m^−^^3^
Monoclinic, *P*2_1_/*n*	Cu *K*α radiation, λ = 1.54184 Å
Hall symbol: -P 2yn	Cell parameters from 2788 reflections
*a* = 5.43804 (19) Å	θ = 3.2–72.4°
*b* = 12.4222 (4) Å	µ = 5.37 mm^−^^1^
*c* = 14.1684 (4) Å	*T* = 173 K
β = 100.269 (3)°	Chunk, colorless
*V* = 941.77 (5) Å^3^	0.26 × 0.12 × 0.06 mm
*Z* = 4	

### Data collection

**Table:**

Agilent Xcalibur (Eos, Gemini) diffractometer	1846 independent reflections
Radiation source: Enhance (Cu) X-ray Source	1650 reflections with *I* > 2σ(*I*))
Graphite monochromator	*R*_int_ = 0.046
Detector resolution: 16.0416 pixels mm^-1^	θ_max_ = 72.5°, θ_min_ = 4.8°
ω scans	*h* = −6→6
Absorption correction: multi-scan (*CrysAlis PRO* and *CrysAlis RED*; Agilent, 2012)	*k* = −12→15
*T*_min_ = 0.691, *T*_max_ = 1.000	*l* = −17→13
5495 measured reflections	

### Refinement

**Table:**

Refinement on *F*^2^	Secondary atom site location: difference Fourier map
Least-squares matrix: full	Hydrogen site location: inferred from neighbouring sites
*R*[*F*^2^ > 2σ(*F*^2^)] = 0.036	H-atom parameters constrained
*wR*(*F*^2^) = 0.100	*w* = 1/[σ^2^(*F*_o_^2^) + (0.0559*P*)^2^ + 0.2711*P*] where *P* = (*F*_o_^2^ + 2*F*_c_^2^)/3
*S* = 1.06	(Δ/σ)_max_ = 0.001
1846 reflections	Δρ_max_ = 0.36 e Å^−^^3^
137 parameters	Δρ_min_ = −0.23 e Å^−^^3^
0 restraints	Extinction correction: *SHELXL97* (Sheldrick, 2008), Fc^*^=kFc[1+0.001xFc^2^λ^3^/sin(2θ)]^-1/4^
Primary atom site location: structure-invariant direct methods	Extinction coefficient: 0.0028 (5)

### Special details

**Table:**

Geometry. All esds (except the esd in the dihedral angle between two l.s. planes) are estimated using the full covariance matrix. The cell esds are taken into account individually in the estimation of esds in distances, angles and torsion angles; correlations between esds in cell parameters are only used when they are defined by crystal symmetry. An approximate (isotropic) treatment of cell esds is used for estimating esds involving l.s. planes.
Refinement. Refinement of *F*^2^ against ALL reflections. The weighted *R*-factor *wR* and goodness of fit *S* are based on *F*^2^, conventional *R*-factors *R* are based on *F*, with *F* set to zero for negative *F*^2^. The threshold expression of *F*^2^ > σ(*F*^2^) is used only for calculating *R*-factors(gt) *etc*. and is not relevant to the choice of reflections for refinement. *R*-factors based on *F*^2^ are statistically about twice as large as those based on *F*, and *R*- factors based on ALL data will be even larger.

### Fractional atomic coordinates and isotropic or equivalent isotropic displacement parameters (Å^2^)

**Table:**

	*x*	*y*	*z*	*U*_iso_*/*U*_eq_	
Cl1	0.35900 (10)	0.10200 (4)	0.67741 (4)	0.02949 (18)	
S1	0.39725 (10)	0.62878 (4)	0.24567 (3)	0.02652 (18)	
N1	0.0469 (3)	0.61595 (14)	0.34925 (13)	0.0253 (4)	
N2	0.2006 (3)	0.52794 (13)	0.36757 (12)	0.0199 (4)	
N3	0.5314 (3)	0.43233 (13)	0.34504 (12)	0.0222 (4)	
C1	0.1320 (4)	0.67447 (17)	0.28675 (15)	0.0261 (4)	
H1	0.0545	0.7386	0.2645	0.031*	
C2	0.3979 (4)	0.51895 (15)	0.32099 (13)	0.0208 (4)	
C3	0.2043 (4)	0.44025 (16)	0.42652 (14)	0.0217 (4)	
H3	0.0929	0.4235	0.4671	0.026*	
C4	0.4101 (4)	0.38271 (15)	0.41207 (13)	0.0190 (4)	
C5	0.5067 (3)	0.28278 (15)	0.46084 (13)	0.0192 (4)	
C6	0.3963 (3)	0.24196 (15)	0.53512 (14)	0.0205 (4)	
H6	0.2605	0.2769	0.5531	0.025*	
C7	0.4911 (4)	0.14891 (16)	0.58176 (13)	0.0213 (4)	
C8	0.6921 (4)	0.09440 (16)	0.55644 (16)	0.0258 (4)	
H8	0.7526	0.0317	0.5881	0.031*	
C9	0.8008 (4)	0.13562 (16)	0.48276 (15)	0.0254 (4)	
H9	0.9360	0.1001	0.4650	0.030*	
C10	0.7107 (4)	0.22930 (15)	0.43499 (14)	0.0219 (4)	
H10	0.7861	0.2563	0.3860	0.026*	

### Atomic displacement parameters (Å^2^)

**Table:**

	*U*^11^	*U*^22^	*U*^33^	*U*^12^	*U*^13^	*U*^23^
Cl1	0.0325 (3)	0.0305 (3)	0.0269 (3)	0.00059 (19)	0.0091 (2)	0.00925 (19)
S1	0.0346 (3)	0.0247 (3)	0.0222 (3)	0.00174 (19)	0.0104 (2)	0.00440 (18)
N1	0.0239 (9)	0.0224 (8)	0.0297 (9)	0.0063 (6)	0.0050 (7)	0.0021 (7)
N2	0.0198 (8)	0.0193 (8)	0.0212 (8)	0.0029 (6)	0.0052 (6)	−0.0002 (6)
N3	0.0228 (8)	0.0234 (8)	0.0221 (8)	0.0031 (7)	0.0087 (7)	0.0004 (6)
C1	0.0292 (11)	0.0235 (10)	0.0246 (10)	0.0033 (8)	0.0019 (8)	0.0003 (8)
C2	0.0243 (10)	0.0224 (9)	0.0167 (9)	−0.0007 (7)	0.0069 (7)	−0.0006 (7)
C3	0.0237 (10)	0.0215 (9)	0.0220 (9)	0.0030 (7)	0.0096 (8)	0.0032 (7)
C4	0.0195 (10)	0.0208 (9)	0.0166 (9)	0.0000 (7)	0.0027 (7)	−0.0023 (7)
C5	0.0190 (9)	0.0200 (9)	0.0181 (9)	−0.0005 (7)	0.0021 (7)	−0.0034 (7)
C6	0.0194 (9)	0.0220 (9)	0.0203 (9)	0.0013 (7)	0.0045 (7)	−0.0021 (7)
C7	0.0212 (10)	0.0228 (9)	0.0197 (9)	−0.0031 (8)	0.0035 (8)	0.0002 (7)
C8	0.0256 (10)	0.0220 (10)	0.0289 (11)	0.0044 (8)	0.0023 (9)	0.0022 (8)
C9	0.0220 (10)	0.0258 (10)	0.0292 (10)	0.0043 (8)	0.0069 (8)	−0.0028 (8)
C10	0.0229 (10)	0.0221 (9)	0.0214 (9)	−0.0003 (8)	0.0063 (8)	−0.0023 (7)

### Geometric parameters (Å, º)

**Table:**

Cl1—C7	1.7432 (19)	C4—C5	1.471 (3)
S1—C2	1.7318 (19)	C5—C6	1.397 (3)
S1—C1	1.744 (2)	C5—C10	1.397 (3)
N1—C1	1.293 (3)	C6—C7	1.385 (3)
N1—N2	1.373 (2)	C6—H6	0.9300
N2—C2	1.361 (3)	C7—C8	1.386 (3)
N2—C3	1.371 (2)	C8—C9	1.386 (3)
N3—C2	1.309 (2)	C8—H8	0.9300
N3—C4	1.393 (2)	C9—C10	1.391 (3)
C1—H1	0.9300	C9—H9	0.9300
C3—C4	1.373 (3)	C10—H10	0.9300
C3—H3	0.9300		
			
C2—S1—C1	87.81 (9)	C6—C5—C10	119.54 (18)
C1—N1—N2	107.27 (17)	C6—C5—C4	119.66 (17)
C2—N2—C3	107.63 (16)	C10—C5—C4	120.79 (18)
C2—N2—N1	118.68 (16)	C7—C6—C5	119.29 (17)
C3—N2—N1	133.69 (17)	C7—C6—H6	120.4
C2—N3—C4	103.49 (15)	C5—C6—H6	120.4
N1—C1—S1	117.63 (16)	C6—C7—C8	121.87 (18)
N1—C1—H1	121.2	C6—C7—Cl1	118.69 (15)
S1—C1—H1	121.2	C8—C7—Cl1	119.41 (16)
N3—C2—N2	112.95 (17)	C9—C8—C7	118.44 (19)
N3—C2—S1	138.46 (15)	C9—C8—H8	120.8
N2—C2—S1	108.59 (14)	C7—C8—H8	120.8
N2—C3—C4	104.29 (16)	C8—C9—C10	121.00 (19)
N2—C3—H3	127.9	C8—C9—H9	119.5
C4—C3—H3	127.9	C10—C9—H9	119.5
C3—C4—N3	111.64 (16)	C9—C10—C5	119.86 (18)
C3—C4—C5	126.96 (18)	C9—C10—H10	120.1
N3—C4—C5	121.37 (17)	C5—C10—H10	120.1
			
C1—N1—N2—C2	0.2 (2)	C2—N3—C4—C3	0.4 (2)
C1—N1—N2—C3	179.7 (2)	C2—N3—C4—C5	−177.68 (17)
N2—N1—C1—S1	0.3 (2)	C3—C4—C5—C6	−4.9 (3)
C2—S1—C1—N1	−0.45 (17)	N3—C4—C5—C6	172.90 (17)
C4—N3—C2—N2	−0.1 (2)	C3—C4—C5—C10	176.33 (19)
C4—N3—C2—S1	179.75 (18)	N3—C4—C5—C10	−5.9 (3)
C3—N2—C2—N3	−0.2 (2)	C10—C5—C6—C7	−0.1 (3)
N1—N2—C2—N3	179.44 (17)	C4—C5—C6—C7	−178.88 (17)
C3—N2—C2—S1	179.87 (13)	C5—C6—C7—C8	−0.4 (3)
N1—N2—C2—S1	−0.5 (2)	C5—C6—C7—Cl1	177.50 (14)
C1—S1—C2—N3	−179.4 (2)	C6—C7—C8—C9	0.5 (3)
C1—S1—C2—N2	0.47 (14)	Cl1—C7—C8—C9	−177.37 (15)
C2—N2—C3—C4	0.5 (2)	C7—C8—C9—C10	−0.1 (3)
N1—N2—C3—C4	−179.12 (19)	C8—C9—C10—C5	−0.4 (3)
N2—C3—C4—N3	−0.6 (2)	C6—C5—C10—C9	0.5 (3)
N2—C3—C4—C5	177.43 (17)	C4—C5—C10—C9	179.27 (18)
